# Cariprazine Augmentation and Sustained Remission of Physical Aggression in Clozapine‐Treated Treatment‐Resistant Schizophrenia: A Case Report

**DOI:** 10.1155/crps/9943329

**Published:** 2026-10-25

**Authors:** Juhani Leijala, Antti Mustonen

**Affiliations:** ^1^ Department of Psychiatry South Ostrobothnia Wellbeing Services County Seinäjoki Finland; ^2^ Department of Psychiatry Faculty of Medicine University of Turku Turku Finland utu.fi; ^3^ Finnish Institute for Health and Welfare (THL) Helsinki Finland thl.fi; ^4^ Faculty of Medicine and Health Technology Tampere University Tampere Finland uta.fi; ^5^ Department of Clinical Neuroscience Karolinska Institutet Stockholm Sweden ki.se

**Keywords:** cariprazine, case report, clozapine, impulsive aggression, schizophrenia, treatment resistance

## Abstract

Persistent aggression may occur in treatment‐resistant schizophrenia (TRS) despite long‐term clozapine treatment and multiple augmentation strategies. We describe a male patient in his sixties with a decades‐long history of schizophrenia who required prolonged psychiatric inpatient care because of recurrent, unpredictable physical assaults toward other patients, staff, relatives, and police. This included treatment at a state forensic psychiatric hospital because of treatment complexity and safety concerns, not forensic legal status. Aggression persisted during adequately monitored high‐dose clozapine treatment and several augmentation strategies. The last documented physical assault occurred in October 2021. Cariprazine 1.5 mg/day was initiated in November 2021, primarily for longstanding negative symptoms. Within approximately seven weeks, mood and participation in communal ward activities improved. No further physical assaults were documented during more than 4 years of follow‐up. The patient subsequently transitioned to voluntary treatment and supported housing. Later somatic illnesses, temporary interruptions of oral medication, and substantial fluctuations in clozapine serum concentrations were not followed by renewed physical aggression. This case describes a prolonged temporal association between cariprazine augmentation and the absence of recurrent physical assaults, but the single‐case design, episodic pretreatment course, concomitant treatment changes, and absence of standardized ratings preclude causal inference.

## 1. Introduction

Treatment‐resistant schizophrenia (TRS) is associated with chronic disability, prolonged inpatient treatment, and high healthcare utilization. Clozapine remains the gold‐standard treatment for TRS and is superior to other antipsychotics in treatment‐refractory populations [1]. It may reduce aggression, hostility, and excitement, but adverse effects and variable exposure require careful clinical and serum‐level monitoring [2, 3]. Partial response to clozapine is common, and residual symptoms, behavioral dysregulation, and aggression may persist despite long‐term treatment and augmentation strategies [4]. Importantly, aggression is not always directly linked to active psychotic symptoms, and management remains challenging [3].

Cariprazine is a dopamine D3/D2 receptor partial agonist with preferential D3 receptor affinity and additional serotonergic activity, including 5‐HT1A partial agonism [5]. It has demonstrated efficacy for predominant negative symptoms in schizophrenia [6] and relapse prevention [7]. Emerging evidence from a prospective series, a retrospective chart review, and a systematic review suggests that cariprazine augmentation may improve negative and other residual symptoms in clozapine‐treated TRS [8, 9, 10]. We report a case in which longstanding recurrent physical aggression did not recur during follow‐up after cariprazine was added to an extensively monitored clozapine‐based regimen.

## 2. Case Presentation

### 2.1. Patient Background and Clinical Course

The patient had been diagnosed with schizophrenia in 1988. In the available electronic medical records, the diagnosis was coded as undifferentiated schizophrenia (ICD‐10 F20.30). The original diagnostic assessment and the rationale for retaining this historical subtype designation were not available for the present retrospective report. The longitudinal clinical picture included disorganization, intermittent auditory hallucinations and paranoid symptoms, episodes of increased fearfulness and withdrawal, prominent negative symptoms, and marked functional impairment. Exacerbations were variably characterized by increased withdrawal, anxiety, disorganization, and psychotic symptoms. The episodes of physical aggression were not consistently associated with an identifiable exacerbation of positive psychotic symptoms. He required supported living for decades and never achieved independent living.

Somatic comorbidities include diabetes mellitus, hyperlipidemia, and psoriasis.

Available electronic laboratory records objectively documented clozapine exposure as early as 1998. The exact date of treatment initiation was unavailable. Subsequent longitudinal records demonstrated long‐term treatment with high doses and repeated serum‐level monitoring, but symptoms and functional impairment remained only partially controlled. After several prolonged admissions and brief unsuccessful residential placements, the patient remained continuously hospitalized from February 2013 until March 2023. Because of recurrent severe and unpredictable violence, treatment complexity, and safety concerns, he spent ~22 months at a state forensic psychiatric hospital before returning to regional psychiatric care in February 2018. The admission was based on treatment complexity and safety concerns, not forensic legal status. The principal clinical events and treatment milestones are summarized in Figure 1.

**Figure 1:**
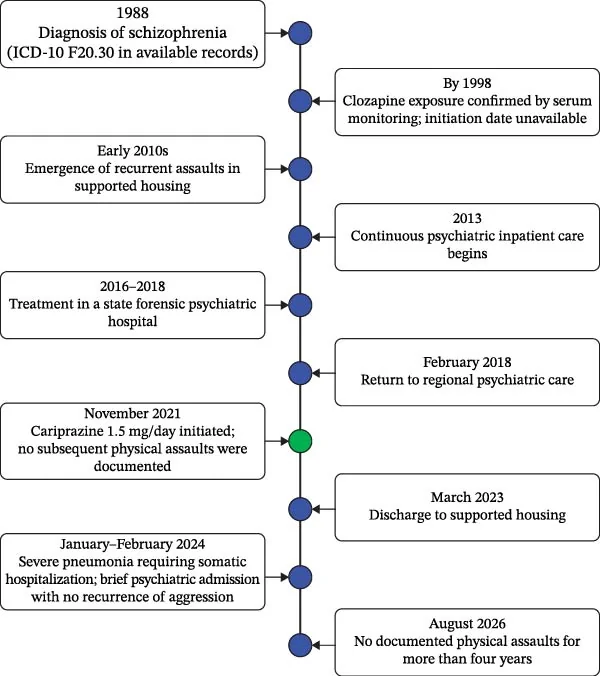
Clinical timeline of illness course and major treatment interventions. The last documented physical assault occurred in October 2021. Cariprazine augmentation was initiated in November 2021, and no further physical assaults were documented during more than 4 years of follow‐up.

### 2.2. Phenomenology of Aggression

Beginning in the early 2010s, the clinical picture became increasingly dominated by recurrent physical aggression across several treatment environments. The assaults were generally sudden and apparently noninstrumental, without consistent prodromal agitation or an immediately identifiable trigger. They were not reliably associated with exacerbations of positive psychotic symptoms. The patient was usually unable to explain the behavior beyond describing an intense, unpleasant feeling and was typically remorseful afterwards. Psychosocial stress, anxiety, and changes in routines nevertheless appeared to increase vulnerability in some episodes.

The course was episodic. A period without documented physical violence from November 2016 until July 2018 ended when the patient abruptly struck a nurse repeatedly during an accompanied walk. Several further assaults occurred during the following weeks. The medication had been administered as prescribed, and no concurrent medication change explained the recurrence. Prominent negative symptoms had been present for years and included marked passivity, social withdrawal, sparse speech, reduced spontaneity and initiative, limited participation in ward activities, and a continuing need for support in daily functioning. Their severity fluctuated and became particularly pronounced after a ward transfer in 2021. However, the records did not demonstrate a consistent temporal relationship between changes in negative symptoms and individual episodes of physical aggression.

### 2.3. History of Pharmacotherapies Prior to Cariprazine Initiation

The patient had a longstanding clinical diagnosis of TRS. The early treatment history predated the TRRIP consensus criteria [11], and the available retrospective records did not contain sufficient dose, duration, adherence, and standardized symptom data to determine whether all TRRIP criteria had been met. Nevertheless, the longitudinal records documented trials of several antipsychotics (chlorpromazine, levomepromazine, perphenazine, olanzapine, quetiapine, amisulpride, and aripiprazole), objectively documented clozapine exposure as early as 1998, subsequent long‐term supervised treatment, exposure to high clozapine doses, repeated therapeutic serum concentrations, and persistent symptoms and marked functional impairment. Mood stabilizers (lithium, valproate, and topiramate), benzodiazepines, and selective serotonin reuptake inhibitors had also been used. We therefore use the descriptive term “clozapine‐treated treatment‐resistant schizophrenia” and do not classify the patient as having formally established clozapine‐resistant schizophrenia.

Immediately before cariprazine initiation, the scheduled regimen consisted of clozapine 575 mg/day, valproate 2000 mg/day, lithium 450 mg/day, quetiapine 75 mg/day with an additional 25 mg as needed, and sertraline 150 mg/day. Clozapine, valproate, lithium, scheduled quetiapine, and sertraline remained unchanged during cariprazine initiation and the early observation period. Amisulpride had been gradually reduced from 400 to 200 mg/day and was discontinued in late October 2021 because of pre‐existing tremor and a temporary medication supply problem. Cariprazine was therefore initiated approximately two weeks after amisulpride discontinuation rather than as a simultaneous cross‐titration.

The patient had spent prolonged periods in psychiatric inpatient care, including continuous hospitalization from February 2013 to March 2023. During the period relevant to cariprazine initiation, all medication was administered under nursing supervision and taken cooperatively without clinically significant refusals. Repeated clozapine serum concentration measurements provided additional objective evidence of medication exposure. Nonadherence was therefore considered an unlikely explanation for persistent symptoms or recurrent aggression. Approximately three weeks before cariprazine initiation, the morning trough serum clozapine concentration at steady state was 1.3 µmol/L, within the therapeutic reference range of ~1.1–1.8 µmol/L. The norclozapine (N‐desmethylclozapine) concentration was 0.4 µmol/L, and the clozapine dose was 575 mg/day. Valproate had been established long before cariprazine initiation; its morning trough serum concentration in June 2021 was 401 µmol/L, within the reference range of ~350–700 µmol/L [12].

### 2.4. Cariprazine Augmentation and Response

The last documented physical assault occurred in October 2021. Cariprazine 1.5 mg/day was initiated in November 2021 primarily to address longstanding negative symptoms, including marked passivity, social withdrawal, reduced spontaneity and initiative, and minimal participation in ward activities. It was not initiated specifically to treat aggression. Approximately seven weeks later, the patient was documented as being in a good mood and spending more time in communal ward areas, and the treating physician considered cariprazine to have had a beneficial effect. At approximately 2 months, his condition was described as stable and calm, with no aggressive behavioral episodes. Because the last documented physical assault occurred before cariprazine initiation, the onset of a possible effect on aggression cannot be determined. The relevant observation was the subsequent prolonged absence of recurrent physical assaults.

Cariprazine was continued at 1.5 mg/day and was not titrated further. Dose escalation was considered, but improvement in mood and communal participation had already been observed at the starting dose. Because the patient also had pre‐existing tremor, a cautious approach was adopted. No akathisia, new or worsening tremor, or other clinically significant adverse effects attributed to cariprazine were documented. Lithium remained at 450 mg/day for approximately 6 months after cariprazine initiation. It was gradually tapered and discontinued in May 2022, primarily because of clinically significant tremor. Recurrent aggression had also continued during earlier lithium treatment, and no aggression recurred after its discontinuation. Valproate, scheduled quetiapine, and sertraline remained unchanged during this period.

No standardized symptom, impulsivity, or aggression rating scales were used. Clinical change was assessed through routine longitudinal observation by the nursing staff and repeated evaluations by treating physicians during inpatient care. The principal behavioral outcome was the presence or absence of documented incidents of physical assault, which were routinely recorded because of their clinical and safety significance. Mood, social withdrawal, participation in communal ward activities, and functional capacity were assessed clinically and documented in the medical record.

### 2.5. Subsequent Course

The patient was discharged from continuous hospitalization in March 2023 to supported housing with outpatient follow‐up. The next available serum clozapine concentrations were 1.8 µmol/L in May 2023 and 1.2 µmol/L in November 2023 (therapeutic reference range: 1.1–1.8 µmol/L), while the clozapine dose remained unchanged. No concentration measurement was available from the early post‐augmentation period. In early 2024, severe COVID‐related pneumonia required somatic hospitalization, followed by psychiatric inpatient care. Oral psychotropic medication, including cariprazine, was temporarily interrupted when oral administration was not possible and was resumed at the previous dose as soon as oral medication became feasible. No physical aggression occurred during or after the interruption.

In 2025, pneumonia was followed by approximately 1 month of mild behavioral deterioration, including unusual behavior and occasional nonaggressive touching of a nearby person’s knee. Clozapine concentrations fluctuated substantially, prompting a temporary dose adjustment. During later follow‐up, including periods of somatic infection, measured serum clozapine concentrations ranged from 0.6 to 3.7 µmol/L. The behavioral symptoms resolved, and no physical aggression was documented. Brief interruptions of cariprazine during later somatic illness likewise reflected the inability to take oral medication rather than adverse effects and were followed by resumption at 1.5 mg/day.

As of August 2026, the patient remained in supported housing under primary care follow‐up, with psychiatric consultation available when required. Treatment included clozapine 500 mg/day, cariprazine 1.5 mg/day, valproate 2000 mg/day, sertraline 150 mg/day, and quetiapine 50 mg/day. Residual functional impairment persisted, and intermittent anxiety, restlessness, and mild disorganized or repetitive behavior had been reported during follow‐up, but no further physical aggression had been documented.

## 3. Discussion

This case describes a prolonged absence of recurrent physical aggression in a patient with clozapine‐treated TRS and prolonged inpatient treatment temporally associated with cariprazine augmentation. Cariprazine was initiated for longstanding negative symptoms rather than aggression. Improved engagement and the subsequent prolonged absence of physical assaults were documented during follow‐up.

Despite decades of supervised clozapine treatment, including high doses and therapeutic serum concentrations, severe physical aggression had continued to recur. In a small case series of six violent patients treated in a high‐security hospital, amisulpride augmentation was associated with fewer aggressive incidents in five patients and improved engagement in all six [13]. Other small uncontrolled studies, including a retrospective study of lurasidone augmentation, have suggested possible clinical benefits in clozapine‐treated patients, although aggression has generally not been the primary outcome [14]. In the present case, prior amisulpride treatment had not prevented recurrent physical aggression. Overall, the available evidence remains insufficient to determine the optimal augmentation strategy for persistent aggression during clozapine treatment.

Previous literature on cariprazine augmentation of clozapine has focused primarily on negative, cognitive, and other residual symptoms rather than aggression [15, 9]. A case report described improvement in autoaggressive compulsive behavior in a patient with TRS and obsessive–compulsive disorder, but this behavior differs phenomenologically from the recurrent impulsive assaults observed in the present case [16]. Findings from bipolar mania studies suggesting benefits of cariprazine on hostility, irritability, and agitation provide only indirect support and cannot be generalized to physical aggression in schizophrenia [17, 18]. Evidence specifically concerning aggression during cariprazine augmentation of clozapine therefore remains sparse and largely uncontrolled.

Aggressive behavior in schizophrenia is heterogeneous and may be related to active psychotic symptoms, instrumental motives, or impulsive and reactive processes [19]. In this case, the assaults were sudden, apparently noninstrumental, and not consistently associated with exacerbations of positive psychotic symptoms. Together with the patient’s subsequent remorse, these features led the episodes to be clinically interpreted as predominantly impulsive. This retrospective interpretation remains uncertain, and contributions from psychotic illness, anxiety, psychosocial stress, and changes in routine cannot be excluded. Cariprazine is a dopamine D3‐preferring D3/D2 partial agonist with additional 5‐HT1A partial agonist activity [5]. One speculative possibility is that this pharmacological profile may have influenced negative symptoms, cognition, or behavioral regulation. However, no neurocognitive, impulsivity, or mechanistic assessments were performed, and neither a direct antiaggressive effect nor a specific D3‐ or 5‐HT1A‐mediated mechanism can be inferred from this case.

Strengths include the long pre‐augmentation history across several treatment environments, supervised medication administration, repeated clozapine serum measurements, contemporaneous clinical documentation, and more than 4 years without further documented physical assaults. Several alternative explanations must, nevertheless, be considered. The patient’s aggression had followed an episodic course, including a previous ~20‐month period without documented physical violence, and natural fluctuation or age‐related stabilization cannot be excluded. The last documented assault preceded cariprazine initiation by several weeks, and the absence of aggression persisted during brief interruptions of cariprazine treatment. Amisulpride had been discontinued approximately two weeks before cariprazine was started, which may have influenced passivity, engagement, or other symptoms. Changes in the ward environment, psychosocial stress, anxiety, residual psychotic and negative symptoms, other concomitant medication, and unmeasured individual or personality‐related factors may also have contributed. The available records did not include a structured personality assessment. Conversely, the principal scheduled medications remained unchanged during the early observation period, medication administration was supervised, and the ward transfer had initially been associated with deterioration rather than improvement. The prolonged temporal association is therefore clinically notable, but the causal inference remains limited.

The absence of standardized PANSS, BPRS, negative‐symptom, impulsivity, or aggression ratings is a major limitation. Outcomes were assessed clinically rather than prospectively, and observer, ascertainment, and documentation bias cannot be excluded. The magnitude of changes in positive, negative, and cognitive symptoms therefore cannot be quantified. A single case cannot establish generalizability or a dose–response relationship. Future prospective case series, observational studies, and, where feasible, controlled trials should use structured measures of psychotic symptoms, negative symptoms, impulsivity, and aggression; document medication exposure and relevant cointerventions; and characterize the phenomenology of aggression prospectively.

## 4. Conclusion

In a patient with clozapine‐treated TRS and longstanding recurrent physical aggression, no further physical assaults were documented after cariprazine 1.5 mg/day was added primarily for negative symptoms. The prolonged temporal association is clinically notable but hypothesis‐generating only. Further prospective observations and controlled studies are needed before any conclusions can be drawn regarding the clinical utility of cariprazine augmentation for persistent aggression.

## Author Contributions

Antti Mustonen and Juhani Leijala contributed to the conception and design of the work and to the interpretation of the case. Juhani Leijala was the responsible physician for the treatment relevant to this report, while Antti Mustonen had been involved in the patient’s earlier care. Both authors drafted the manuscript and revised it critically for important intellectual content.

## Funding

This study was funded by the Juho Vainio Foundation, State Funding for University‐Level Health Research, Tampere University Hospital and the Finnish Foundation for Alcohol Studies.

## Disclosure

Antti Mustonen and Juhani Leijala affirm that the manuscript is an honest, accurate, and transparent account of the study being reported. No important aspects of the study have been omitted and any discrepancies from the study as planned have been explained. Both authors have approved the final version. All AI‐assisted suggestions were reviewed, revised, and approved by the authors, who take full responsibility for the content of the manuscript. The funders of the study had no role in study design, data collection, data analysis, data interpretation, or writing of the report.

## Ethics Statement

According to the institutional determination of the Wellbeing Services County of South Ostrobothnia, this retrospective single‐patient case report, describing treatment and events occurring during routine clinical care, did not require institutional research permission or review by a research ethics committee. No interventions or procedures were performed for research or publication purposes. All potentially identifying information has been omitted to protect the patient’s privacy.

## Consent

Written informed consent for publication of this case report was provided by the patient’s legally authorized representative using the publisher’s consent form. The patient was also informed about the planned publication and verbally agreed to it. A copy of the written consent is available for review by the Editor‐in‐Chief upon request.

## Conflicts of Interest

Juhani Leijala has received research funding or grants from the Wellbeing Services County of South Ostrobothnia, State Funding for University‐Level Health Research, the Finnish Medical Foundation, the Finnish Psychiatric Association, and the Psychiatric Research Foundation. Juhani Leijala has received lecture fees from Orion Pharma, Otsuka, and Lundbeck. Antti Mustonen has received funding from the Juho Vainio Foundation, the Wellbeing Services County of South Ostrobothnia, State Funding for University‐Level Health Research, Tampere University Hospital, and the Finnish Foundation for Alcohol Studies. Antti Mustonen has received lecture fees from Recordati, Lundbeck, and Otsuka; advisory board compensation from Recordati; and travel and congress support from Recordati.

## Patient Perspective

Because of limited illness insight and longstanding cognitive and negative symptoms, the patient was unable to provide a detailed medication‐specific perspective. He nevertheless regarded his medication as beneficial and, during follow‐up, reported a good mood and satisfaction with his supported living environment.

## Data Availability

The clinical data underlying this case report are contained in confidential medical records and cannot be made publicly available because of patient privacy and legal restrictions. Relevant deidentified information is presented within the article.
